# Recent advances in oridonin derivatives with anticancer activity

**DOI:** 10.3389/fchem.2023.1066280

**Published:** 2023-02-09

**Authors:** Pedro J. M. Sobral, André T. S. Vicente, Jorge A. R. Salvador

**Affiliations:** ^1^ Laboratory of Pharmaceutical Chemistry, Faculty of Pharmacy, University of Coimbra, Coimbra, Portugal; ^2^ Center for Neuroscience and Cell Biology, University of Coimbra, Coimbra, Portugal

**Keywords:** oridonin, diterpenoid derivatives, anticancer activity, drug discovery, structural modification

## Abstract

Cancer is a leading cause of mortality responsible for an estimated 10 million deaths worldwide in 2020, and its incidence has been rapidly growing over the last decades. Population growth and aging, as well as high systemic toxicity and chemoresistance associated with conventional anticancer therapies reflect these high levels of incidence and mortality. Thus, efforts have been made to search for novel anticancer drugs with fewer side effects and greater therapeutic effectiveness. Nature continues to be the main source of biologically active lead compounds, and diterpenoids are considered one of the most important families since many have been reported to possess anticancer properties. Oridonin is an *ent-*kaurane tetracyclic diterpenoid isolated from *Rabdosia rubescens* and has been a target of extensive research over the last few years. It displays a broad range of biological effects including neuroprotective, anti-inflammatory, and anticancer activity against a variety of tumor cells. Several structural modifications on the oridonin and biological evaluation of its derivatives have been performed, creating a library of compounds with improved pharmacological activities. This mini-review aims to highlight the recent advances in oridonin derivatives as potential anticancer drugs, while succinctly exploring their proposed mechanisms of action. To wind up, future research perspectives in this field are also disclosed.

## 1 Introduction

Cancer is a devastating disease. Based on the most recent estimates of global mortality and incidence data (2020), cancer was responsible for 10 million deaths and 19.3 million new cases worldwide. Growth and aging of the population, in most countries, and changes in the distribution of the main risk factors, are some reasons that explain these high levels of mortality and incidence, which are not predicted to decrease in the coming years (Bray et al., 2021; Sung et al., 2021).

There is no curative treatment option available for cancer, and current anticancer therapies, especially chemotherapy, have limited therapeutic potential associated with adverse side effects, chemoresistance, and high systemic toxicity to the patient (Kashyap et al., 2021). Consequently, studies have been performed to search for more efficient and selective anticancer drugs with greater therapeutic properties and better safety profiles. Over the last few years, there has been a growing attention toward the development of natural anticancer agents.

Natural products are recognized as important sources of lead compounds, characterized not only by their remarkable biological activity, but also by their diverse and complex structures. Since natural products are produced by living organisms, they possess properties that are evolutionarily optimized for serving a biological function, such as binding to a specific macromolecule (Mayack et al., 2020). These attributes invite researchers to make structural modifications and optimizations, in search of novel natural product derivatives. Between 1981 and 2019, a detailed analysis of all therapeutic agents approved revealed that about 60% of the currently used anticancer drugs came from natural products (Newman and Cragg, 2020). Therefore, they continue to hold great potential in the search for novel lead compounds in drug discovery, especially in anticancer therapy.

Diterpenoids are considered one of the most important families of natural products. Oridonin is an *ent-*kaurane diterpenoid that has attracted an increasing amount of attention in recent years, due to its extensive biological activities (Ding et al., 2016). Despite oridonin’s remarkable anticancer activity, its potential clinical use is limited. Therefore, researchers have structurally modified oridonin and synthesized new derivatives with improved pharmacological activities and drug-like properties (Liu et al., 2021b).

Herein we seek to briefly overview the biological activities of oridonin and highlight the emerging therapeutic potential of recent oridonin derivatives in anticancer therapy, while also exploring their proposed mechanisms of action. Finally, we provide a discussion of future research perspectives for the development of these derivatives in the clinic.

## 2 Oridonin: An active compound with anticancer activity

Oridonin (C_20_H_28_O_6_, compound 1 listed in Table 1) is an *ent-*kaurane tetracyclic diterpenoid isolated from the traditional Chinese medicinal herb *Rabdosia rubescens*. It was first reported in 1967 (Fujita et al., 1967), and the relationship between its anticancer activity and its structure was demonstrated a few years later (Fujita et al., 1976).

**TABLE 1:** Recent approaches of oridonin optimizations (modifications of hydroxyl groups).

No.	Chemical structure	*In vitro* activity (µM)	References
1	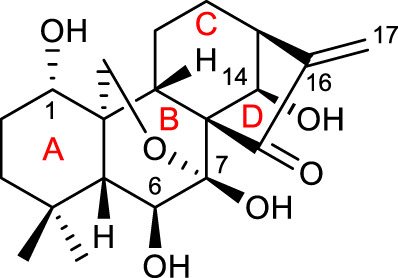	HL-60 IC_50_ = 32.76	Xu et al., 2008, 2016a, 2017, Wang et al. (2011), Ding et al. (2013a), Hou et al. (2019), Shen et al. (2019), Li et al. (2020), Yao et al. (2020)
		BEL-7402 IC_50_ = 39.80	
		BGC-7901 IC_50_ = 28.30	
		HCT-116 IC_50_ = 6.84	
		PC-3 IC_50_ = 13.9	
		K562 IC_50_ = 4.57	
		HCC-1806 IC_50_ = 21.74	
		MCF-7 IC_50_ = 17.9	
		MDA-MB-231 IC_50_ = 29.40	
		Growth inhibitory rate (BEL-7402)	
		29.9% (1 µM)	
		93,6% (10 µM)	
		*In vivo* activity	
		Tumor inhibitory ratio (H22): 42.7%	
		Tumor inhibitory ratio (B16): 45.9%	
2	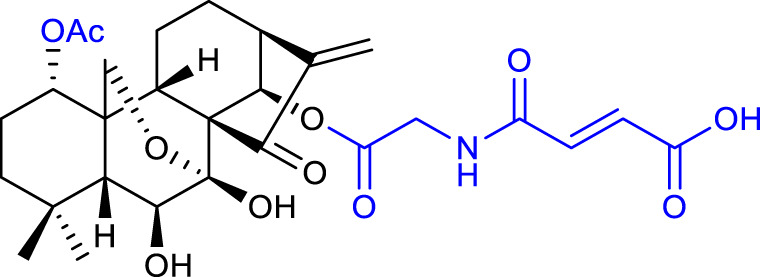	HL-60 IC_50_ = 0.84	Xu et al. (2008)
		BGC-7901 IC_50_ = 2.78	
		*In vivo* activity	
		Tumor inhibitory ratio (H22): 64.9%	
		Tumor inhibitory ratio (B16): 69.9%	
3	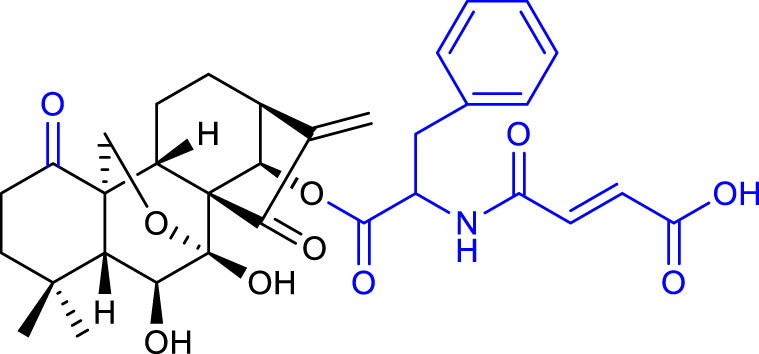	BEL-7402 IC_50_ = 1.00	Xu et al. (2008)
		BGC-7901 IC_50_ = 3.02	
		*In vivo* activity	
		Tumor inhibitory ratio (H22): 62.5%	
		Tumor inhibitory ratio (B16): 61.2%	
4	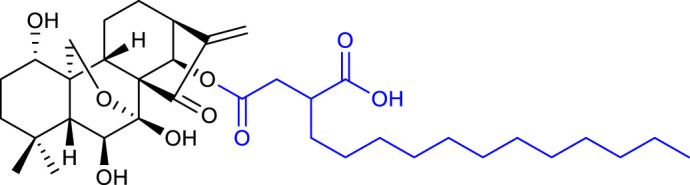	HL-60 IC_50_ = 4.21	Wang et al. (2011)
		BGC-7901 IC_50_ = 1.05	
		*In vivo* activity	
		Tumor inhibitory ratio (H22): 63.7%	
5	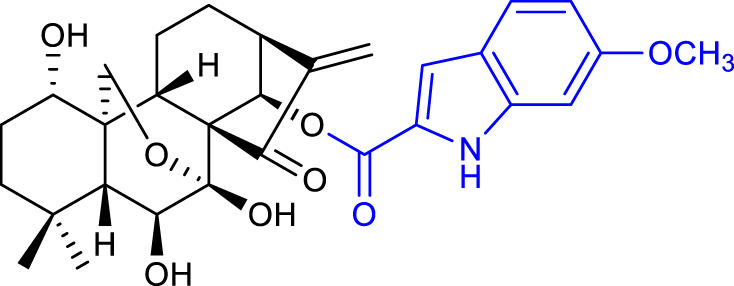	BEL-7402 IC_50_ = 2.18	Shen et al. (2019)
		HCT-116 IC_50_ = 0.16	
6	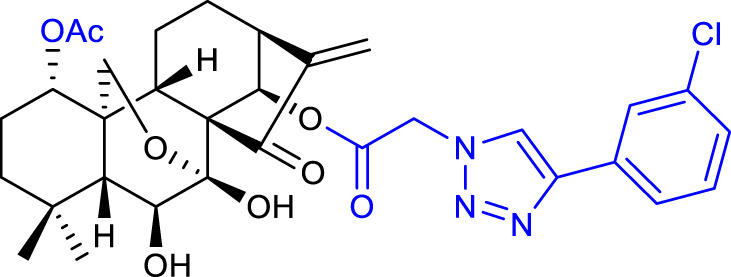	HCT-116 IC_50_ = 32.4	Hou et al. (2019)
		PC-3 IC_50_ = 3.1	
		K562 IC_50_ = 11.1	
7	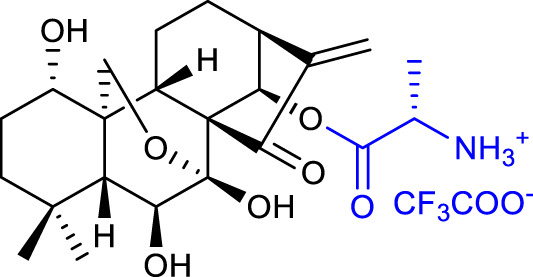	*data not disclosed*	Sun et al. (2014)
8	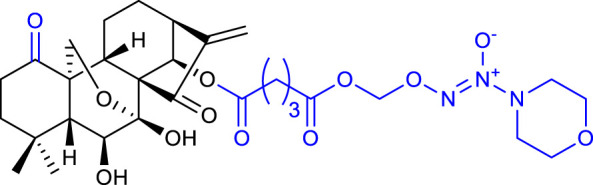	BEL-7402 IC_50_ = 1.84	Xu et al. (2016a)
		Growth inhibitory rate (BEL-7402)	
		87,77% (1 µM)	
		93,94% (10 µM)	
9	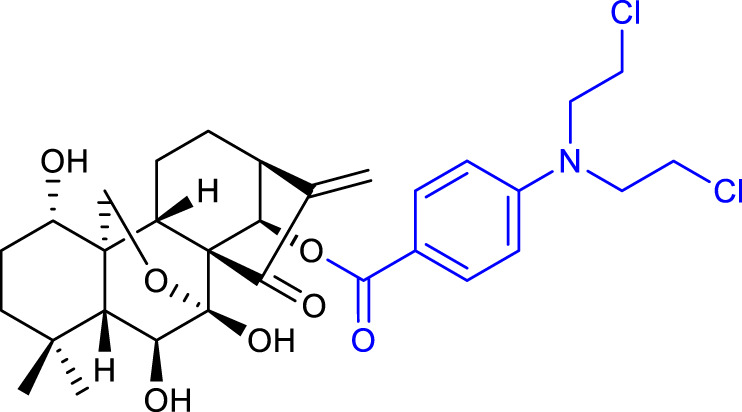	BEL-7402 IC_50_ = 0.50	Xu et al. (2014)
		K562 IC_50_ = 1.12	
10	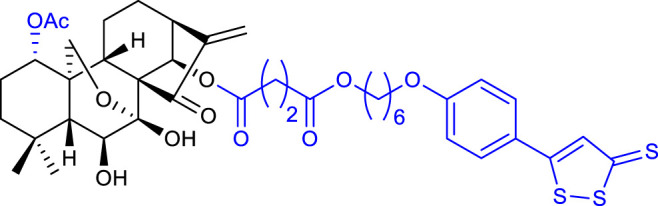	K562 IC_50_ = 0.95	Li et al. (2020)
		MCF-7 IC_50_ = 16.15	
11	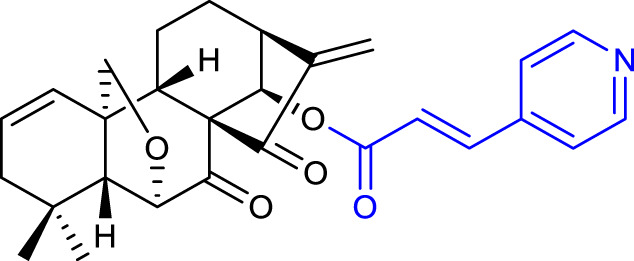	HCC-1806 IC_50_ = 0.18	Yao et al. (2020)
		MDA-MB-231 IC_50_ = 0.63	

Besides its anticancer activity, oridonin possesses an extensive range of biological activities, such as anti-inflammatory (Cummins et al., 2019), neuroprotective (Lin et al., 2019), anti-microbial (Li et al., 2016b), anti-fibrotic (Bohanon et al., 2014), anti-sepsis (Zhao et al., 2016), immune-modulating (Guo et al., 2013), and analgesic effects (Zang et al., 2016). A search of the PubMed.gov 0F^1^ database revealed the following results: 617 research articles published as of September 2022 while searching “oridonin”, and 357 research articles published as of September 2022 while searching “oridonin AND cancer”. This indicates that a major focus is being given to its anticancer activity.

Oridonin’s anticancer activity is well documented in a variety of cancers, in particular, lung (Li et al., 2018b), prostate (Lu et al., 2017), esophageal (Jiang et al., 2019), liver (Zhang et al., 2006), colorectal (Zhang et al., 2019a), breast (Li et al., 2018a), gastric (He et al., 2017), pancreatic (Liu et al., 2020), oral (Yang et al., 2017), nasopharyngeal (Liu et al., 2021a), gallbladder (Chen et al., 2019), ovarian (Dong et al., 2018), leukemia (Li and Ma, 2019), and myeloma (Hong et al., 2020).

Nevertheless, oridonin’s anticancer mechanisms of action are not yet fully understood. The suggested main ones include the suppression of the cell cycle progression, induction of apoptosis, and autophagy, by modulation of signaling pathways, for instance, regulation of intracellular reactive oxygen species (ROS), Bax/Bcl-2, p53/p21, NF-κB, MAPK, PI3K, and fatty acid synthase pathways (Ding et al., 2016).

**FIGURE 1:**
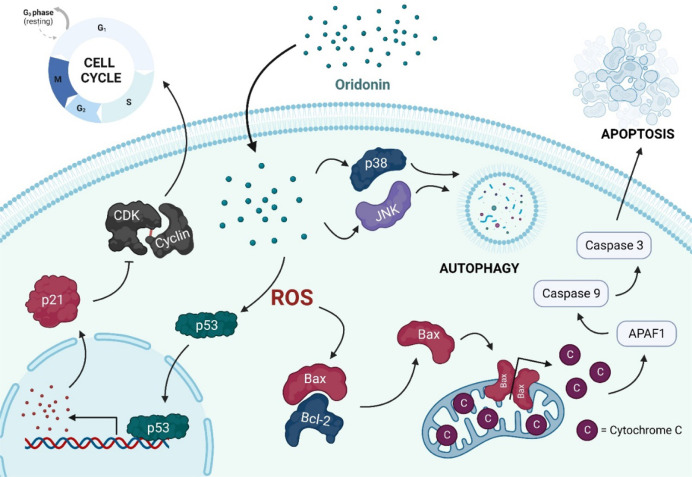
Schematic representation of the relevant signaling pathways modulated by oridonin.

The relevant signaling pathways modulated by oridonin are represented in Figure 1.

As documented in the literature, oridonin induced G2/M phase arrest of A549 cells (Zheng et al., 2017) and promoted S phase arrest *via* the p53/p21 pathway on activated hepatic stellate cells (Bohanon et al., 2014). Oridonin also inhibited the proliferation and induced apoptosis of SNU-216 cells by enhancing the p53 expression and function (Bi et al., 2018), and increased the generation of ROS, triggering apoptosis, in diffuse large B-cell lymphoma (Xu et al., 2016b).

Moreover, oridonin induced apoptosis in OCM-1 and MUM2B uveal melanoma cells (Gu et al., 2015), and SW480 and SW620 colorectal cancer cells (Kwan et al., 2013), by suppressing fatty acid synthase. In HepG2 cells, oridonin induced G2/M phase arrest and apoptosis *via* MAPK and p53 pathways (Wang et al., 2010).

Furthermore, oridonin also induced autophagy by inhibiting glucose metabolism in colorectal cancer cells (Yao et al., 2017), and recent studies suggest that oridonin not only can suppress cell migration and invasion, (Li et al., 2016c), but also revert drug resistance (Kadioglu et al., 2018).

## 3 Oridonin derivatives: Potential agents in anticancer therapy

Oridonin is recognized as a logical hit compound for anticancer therapy research. It has an appropriate molecular weight (364.4 g/mol) and plenty of functional groups that provide numerous synthetic routes to create different libraries of derivatives. Oridonin also meets the criteria of Lipinski’s rule (Lipinski et al., 2001) and it is relatively commercially available (Ding et al., 2016).

However, oridonin’s use as a therapeutic agent is limited by its low water solubility and oral bioavailability (Xu et al., 2006; Xu et al., 2018), as well as its first-pass effect after oral administration. Moreover, its rapid clearance, lack of proper dosage forms, moderate potency, and still undefined mechanisms of action (Li et al., 2016a) also limit oridonin’s use in the clinic. Therefore, a main strategy to overcome such shortcomings is by synthesizing oridonin derivatives with increased drug-likeness properties and anticancer activity (Xu et al., 2018).

**FIGURE 2:**
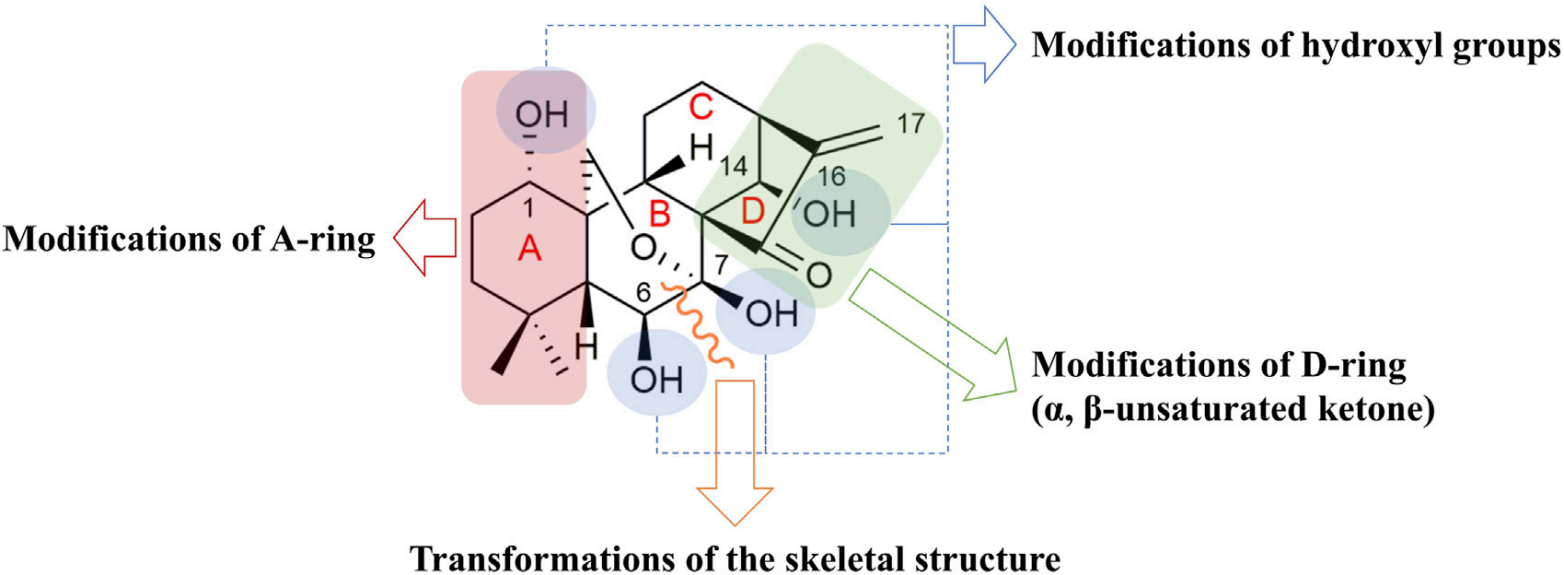
Typical optimization sites and molecular structure of oridonin.

Evidence shows that structural modifications on oridonin usually encompass four typical optimizations, as represented in Figure 2: 1) modifications of hydroxyl groups; 2) modifications of A-ring; 3) modifications of D-ring (α, β-unsaturated ketone); and 4) transformations of the skeletal structure (Zhang et al., 2020; Guan et al., 2021). Although this research has been active for a few years, and substantial progress has been achieved in the identification of novel derivatives, herein we have selected the most representative work.

### 3.1 Modifications of hydroxyl groups

Xu et al. synthesized a series of oridonin derivatives by introducing various hydrophilic side chains at the 1-*O*- and 14-*O*-hydroxyl groups. Most of them exhibited improved cytotoxicity and aqueous solubility. Compound 2 (listed in Table 1) is almost 38-fold more potent than oridonin in the HL-60 cell line (IC_50_ = 0.84 μM), and compound 3 (listed in Table 1) is almost 40-fold more potent than oridonin in the BEL-7402 cell line (IC_50_ = 1.00 μM). *In vivo*, compounds 2 and 3 showed a more potent anticancer effect in mice with H22 liver tumor (tumor inhibitory ratio of 64.9% and 62.5%, respectively) and in mice with B16 melanoma (tumor inhibitory ratio of 69.9% and 61.2%, respectively) when compared with its parental compound (Xu et al., 2008).

A follow-up paper was published regarding the conjugation of different anhydrides with the 14-*O-*hydroxyl group and further reaction with an amino acid ester. Amino acid modifications can be performed to improve the compound’s solubility and cell permeability (Vig et al., 2013). The results afforded compound 4 (listed in Table 1), with an anticancer activity almost 27-fold more potent against the BGC-7901 cell line (IC_50_ = 1.05 μM) and a tumor inhibitory ratio of 63.7% in mice with H22 liver tumor when compared with oridonin (Wang et al., 2011).

In 2019, Shen et al. also synthesized oridonin derivatives by modifying the 14-*O-*hydroxyl group. Compound **5** (listed in Table 1) proved to be the most potent (IC_50_ = 0.16 μM), around 43-fold more potent than oridonin against the HCT-116 cell line. Moreover, this compound induced cell cycle arrest at the S and G2/M phases, and apoptosis progression, possibly by suppressing the p53-MDM2 signaling pathway. Furthermore, *in vivo* studies on an HCT-116 colon cancer xenograft model reported compound 5 to suppress the tumor volume and reduce its weight by 85.82% at 25 mg/kg/day, when compared with oridonin (58.61%) (Shen et al., 2019).

In the same year, Hou et al. synthesized novel C14-1,2,3-triazole oridonin derivatives *via* copper-catalyzed alkyne-azide cycloaddition (CuAAC). Compound 6 (listed in Table 1) proved to be the most potent (IC_50_ = 3.1 μM) against the PC-3 cancer cell line. Preliminary mechanistic studies reported that this compound caused G2/M phase arrest and induced apoptosis in a dose-dependent manner in the same cell line (Hou et al., 2019).

A major milestone was achieved when Sun and collaborators synthesized L-alanine-(14-oridonin) ester trifluoroacetate (HAO472) (compound **7**, listed in Table 1). The compound exhibited improved aqueous solubility without losing anticancer activity (data not disclosed). *In vivo*, HAO472 acts as a prodrug, releasing oridonin when metabolized through the cleavage of its C14 ester bond. HAO472 advanced into a phase I human clinical trial (CTR20150246; chinadrugtrials. org.cn)1F^2^ in China, by Jiangsu Hengrui Medicine Co., Ltd., to develop a new treatment for acute myelogenous leukemia (Sun et al., 2014).

Xu’s group synthesized novel derivatives possessing NO donor functionalities with modifications at the 1-*O-* and 14-*O-*hydroxyl groups. Compound 8 (listed in Table 1) showed the most potent anticancer activity against the BEL-7402 cell line (IC_50_ = 1.84 μM). Preliminary mechanistic studies revealed that compound 8 induced apoptosis and caused S phase arrest in BEL-7402 cells, exhibiting a growth inhibitory rate of 87.7% and 93.9% for 1 μM and 10 μM respectively when compared with oridonin (Xu et al., 2016a).

The same research group also synthesized oridonin-coupled nitrogen mustard derivatives, and all of them showed better anticancer activity than oridonin against a variety of cell lines. Compound 9 (listed in Table 1) proved to be the most potent, exhibiting an IC_50_ value of 0.50 μM against the BEL-7402 cell line. This compound also induced apoptosis of BEL-7402 cells and caused G1 phase arrest (Xu et al., 2014).

In 2020, Li and collaborators synthesized oridonin derivatives with H_2_S-releasing groups. Compound 10 (listed in Table 1) showed the most potent anticancer activity against the K562 cell line, with an IC_50_ value of 0.95 μM. Further studies revealed that compound 10 caused S phase arrest in K562 cells and G1 phase arrest in HepG2 cells (Li et al., 2020).

In the same year, Yao and collaborators synthesized oridonin derivatives by eliminating all hydroxyl groups of oridonin. Compound 11 (listed in Table 1) exhibited an IC_50_ value of 0.18 μM against the HCC-1806 cell line, 120-fold more potent than oridonin. Moreover, this compound induced ROS generation, caused G2/M phase arrest and induced apoptosis through the PI3K-Akt-mTOR signaling pathway. Furthermore, *in vivo* studies in mice with breast cancer reported that compound 11 suppressed tumor volume and reduced its weight by 74.1% at 25 mg/kg/day, which was better than the positive control paclitaxel (66.0% at 6 mg/kg/day) while showing no toxicity (Yao et al., 2020).

The reported structural modifications generally improve the solubility of the derivatives by introducing aqueous solubility-enhancing moieties *via* esterification of the 1-*O* and 14-*O*-hydroxyl groups. Such derivatives usually act as prodrugs since ester bonds suffer from poor *in vivo* metabolic stability (Ding et al., 2013a).

### 3.2 Modifications of A-ring

In 2017, Xu’s group synthesized and evaluated a panel of A-ring modified derivatives bearing various substituents on the 14-*O-*position. The results indicated that the anticancer efficacy was highly dependent on the 14-position modification and the 1-*O-*hydroxyl group was not required for efficacy. Compound 12 (listed in Table 2), with a *trans-*cinnamic acid moiety on the 14-position, displayed the most potent activity against the MCF-7 cell line with an IC_50_ value as low as 0.08 μM, 200-fold more potent than oridonin. Moreover, compound 12 caused ROS generation, induced apoptosis *via* the mitochondrial pathway, and arrested the cell cycle at the G2/M phase (Xu et al., 2017).

**TABLE 2:** Recent approaches of oridonin optimizations (modifications of A-ring).

No.	Chemical structure	*In vitro* activity (µM)	References
12	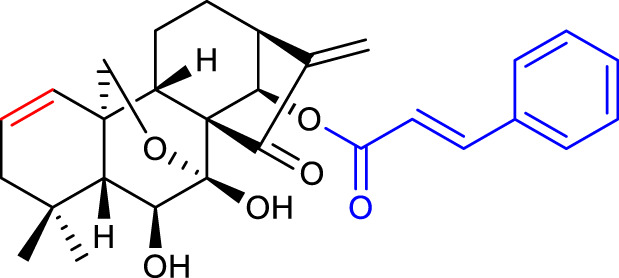	BEL-7402 IC_50_ = 1.03	Xu et al. (2017)
		K562 IC_50_ = 0.29	
		MCF-7 IC_50_ = 0.08	
13	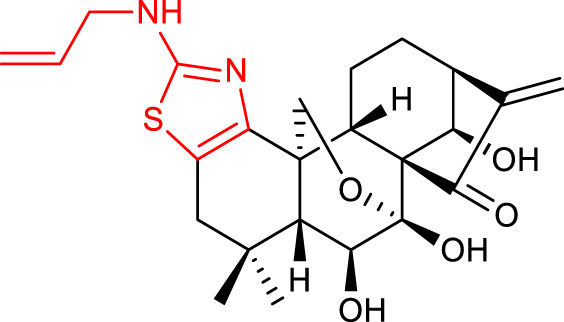	MCF-7 IC_50_ = 0.20	Ding et al. (2013a)
		MDA-MB-231 IC_50_ = 0.20	
14	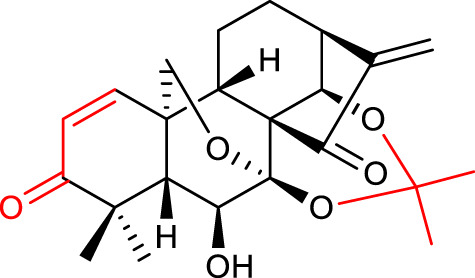	MCF-7 IC_50_ = 0.98	Ding et al. (2013b)
		MDA-MB-231 IC_50_ = 5.60	
15	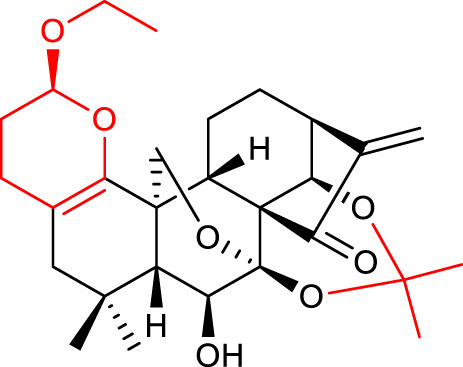	MCF-7 IC_50_ = 0.44	Ding et al. (2014)
		MDA-MB-231 IC_50_ = 0.54	

In 2013, Ding et al. (2013a) developed novel derivatives by introducing a thiazole ring at C1 and C2 of oridonin’s A-ring. Most of the nitrogen-enriched derivatives exhibited higher potency and aqueous solubility. In the form of its HCl salt, compound 13 (listed in Table 2) exhibited approximately 62-fold improvement in aqueous solubility when compared with oridonin (1.29 mg/mL). Additionally, being the most potent, compound 13 showed an IC_50_ value of 0.20 μM, approximately 147-fold more potent than oridonin, and mediated apoptosis of MDA-MB-231 cells.

The enone and pyran systems are important functionalities naturally occurring in various bioactive compounds (Kumar et al., 2017). Ding et al. (2013b) synthesized novel derivatives by incorporating them into the A-ring of oridonin. The introduction of the enone functionality created dienone derivatives, and compound 14 (listed in Table 2) proved to be the most promising with an IC_50_ value of 0.98 μM against the MCF-7 cell line, inducing apoptosis of MCF-7 cells by inhibiting NF-κB pathway and increasing Bax/Bcl-2 ratio. Among the dihydropyran-fused derivatives, compound 15 (listed in Table 2) showed the highest inhibition potency against the same cell line (IC_50_ = 0.44 μM), but no mechanistic studies were provided for this compound (Ding et al., 2014).

### 3.3 Modifications of D-ring (α, β-unsaturated ketone)

α, β-unsaturated ketones (enones) are well-known Michael acceptors. For this reason, the enone system is considered an important pharmacophore of natural products, and oridonin’s D-ring enone appears to be critical for its anticancer activity (Ding et al., 2013b). Hence, few modifications have been performed on that part of the molecule that have successfully produced derivatives with improved activity.

Nonetheless, Shen et al. (2018) demonstrated that α, β-unsaturated ketones can be targets for structural modifications to achieve promising derivatives. Shen’s group synthesized oridonin derivatives with substituted benzene moieties at the C17 position, and compound 16 (listed in Table 3) proved to be the most potent with an IC_50_ value of 1.05 μM against the HCT-116 cell line. Moreover, compound **16** induced apoptosis and caused G2 phase arrest in HCT-116 cells.

**TABLE 3:** Recent approaches of oridonin optimizations (modifications of D-ring - α, *β*-unsaturated ketone).

No.	Chemical structure	*In vitro* activity (µM)	Reference
16	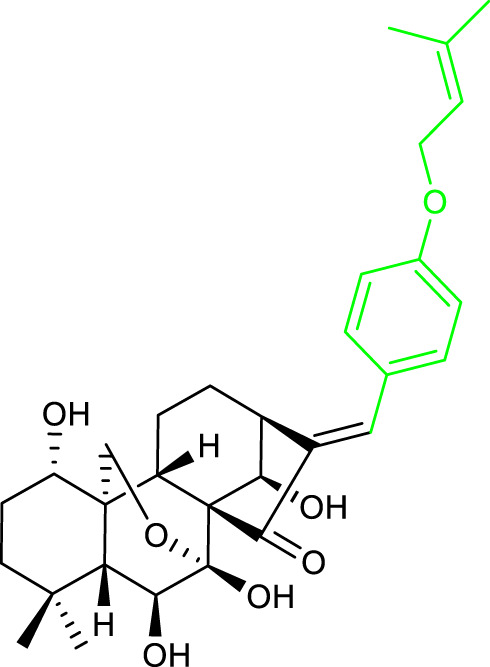	BEL-7402 IC_50_ = 3.21	Shen et al. (2018)
		HCT-116 IC_50_ = 1.05	

### 3.4 Transformations of the skeletal structure

6,7-seco oridonin derivatives (especially spirolactone-type and enmein-type diterpenoids) have been reported to possess impressive anticancer activity. Unfortunately, they are harder to isolate from natural plant sources than oridonin. Since oridonin is commercially available, it can be used as a starting material to synthesize these compounds: the C6-C7 carbon bond of oridonin can be oxidized and cleaved in the presence of periodate or lead tetraacetate, yielding a 6,7-seco-kaurene-type diterpenoid. If the starting material has a hydroxyl group at C1, an enmein-type is obtained; otherwise, a spirolactone-type is formed (Wang et al., 2012; Ding et al., 2016; Xu et al., 2018).

Li et al. (2013a) synthesized *ent-*6,7-*seco-*oridonin derivatives by the conversion of oridonin to spirolactone-type diterpenoids. All the synthesized compounds exhibited better anticancer activity than oridonin, *in vitro*. Compound 17 (listed in Table 4) exhibited IC_50_ values of 0.39 μM against the K562 cell line and 1.39 μM against the BEL-7402 cell line, similar values to that of the positive control Taxol (IC_50_ values of 0.41 μM and 1.89 μM, respectively). Further mechanistic studies of compound 17 revealed that it induced apoptosis in BEL-7402 cells and caused G2/M phase arrest.

**TABLE 4:** Recent approaches of oridonin optimizations (transformations of the skeletal structure).

No.	Chemical structure	*In vitro* activity (µM)	References
17	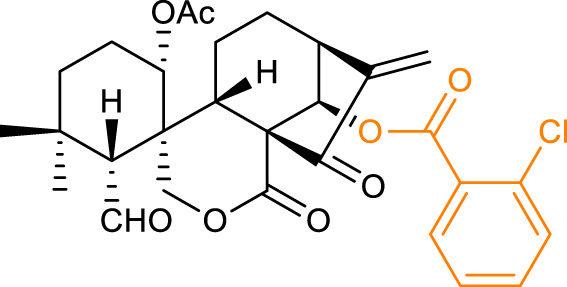	BEL-7402 IC_50_ = 1.39	Li et al. (2013a)
		K562 IC_50_ = 0.39	
18	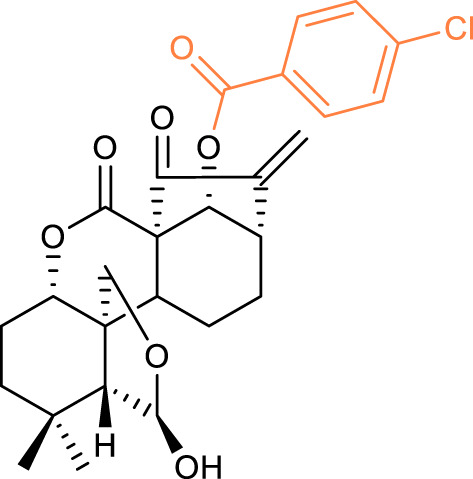	BEL-7402 IC_50_ = 0.87	Li et al. (2013b)
		K562 IC_50_ = 0.24	

The same research group reported a series of novel enmein-type derivatives, and most of them exhibited improved anticancer activities when compared with oridonin and the positive control Taxol. The representative compound 18 (listed in Table 4) showed IC_50_ values of 0.24 μM against the K562 cell line and 0.87 μM against the BEL-7402 cell line. Moreover, compound 18 caused G2/M phase arrest and induced apoptosis by triggering the mitochondria-related caspase-dependent pathway (Li et al., 2013b).

## 4 Future perspectives

The global cancer burden is expected to be 28.4 million cases in 2040, corresponding to a 47% rise from 2020 (Sung et al., 2021).

Chemotherapy continues to be the main therapeutic option for cancer treatment, and oridonin has recently emerged as a promising hit compound due to its anticancer activity. However, its therapeutic potential is limited, and the exact mechanisms of action remain to be further elucidated.

Tremendous efforts to improve oridonin’s pharmaceutical properties have been carried out by several research groups. To date, over one hundred oridonin-based new scaffolds with various modifications have been synthesized, and many of them exhibited improved anticancer activities and aqueous solubility (Guan et al., 2021). Moreover, the structure-activity relationship studies obtained have contributed to a better comprehension of their mechanisms of action and molecular targets (Li et al., 2021).

Oridonin has also been investigated in combination therapy with other chemotherapeutic agents. For instance, oridonin was shown to potentiate the apoptotic effects of gemcitabine through G0/G1 phase arrest in the PANC-1 cell line (Liu et al., 2014), and synergistically enhance JQ1-triggered apoptosis in HCC cells through the mitochondrial pathway (Zhang et al., 2017). Combined treatment of oridonin with cetuximab showed synergistic anticancer effects on laryngeal squamous cell carcinoma (Cao et al., 2016). Moreover, oridonin and homoharringtonine (HHT) exerted synergistic effects against t (8; 21) leukemia *in vitro* and *in vivo* prolonging t (8; 21) leukemia mouse survival (Zhang et al., 2019b). Furthermore, a reported study demonstrated that oridonin exhibited anti-chemoresistance activity in cisplatin-resistant human gastric cancer cells by inducing caspase-dependent apoptosis (He et al., 2017). Altogether, these findings lead us to believe that oridonin and its derivatives have tremendous potential yet to be discovered in a variety of cancers, either in single or combined therapy.

Although no oridonin-based drugs have been approved for clinical use by the U.S. Food and Drug Administration (FDA) or by the European Medicines Agency (EMA) (Liu et al., 2021b), compound HAO472 has already advanced into a phase I clinical trial in China, and we anticipate that new oridonin derivatives may emerge as anticancer drug candidates and enter additional clinical trials soon. It is imperative for oridonin and its derivatives to be the subjects of more robust pre-clinical studies, to ensure the safety and potency of the compounds before developing them as anticancer drugs.

Further investigations are required regarding the single or combined use of oridonin and its derivatives in anticancer therapy, while also exploring their role as anti-chemoresistance agents, for they have the potential to be viable therapeutic options.

## Footnotes

1
https://pubmed.ncbi.nlm.nih.gov/

2
http://www.chinadrugtrials.org.cn/index.html
